# Vitamin D receptor gene polymorphisms in association with diabetic nephropathy: a systematic review and meta-analysis

**DOI:** 10.1186/s12881-017-0458-8

**Published:** 2017-08-29

**Authors:** Lina Yang, Lan Wu, Yi Fan, Jianfei Ma

**Affiliations:** 1 Department of Geriatrics, the First Affiliated Hospital of China Medical University, 155th Nanjing North Street, Shenyang, Liaoning 110001 People’s Republic of China; 2 Department of Nephrology, the First Affiliated Hospital of China Medical University, 155th Nanjing North Street, Shenyang, Liaoning 110001 People’s Republic of China

**Keywords:** Vitamin D receptor, Gene polymorphisms, Diabetic nephropathy, Meta-analysis

## Abstract

**Background:**

A large amount of researches have demonstrated that vitamin D receptor (VDR) gene polymorphisms are associated with diabetic nephropathy (DN) risk in diabetes mellitus (DM) patients. Nevertheless, the results are inconclusive and inconsistent.

**Methods:**

We screened PubMed, Embase, Chinese National Knowledge Infrastructure and Chinese Wanfang databases for those relevant studies updated in May 2016.

**Results:**

7 studies involving 2564 subjects were recruited. We evaluated the genotypic and allelic differences between DN patients and DM controls. Overall analysis showed that no significant association was found among the ApaI, BsmI, FokI,TaqI gene polymorphisms and DN susceptibility in diabetic patients (all *P* values > 0.05). In the stratified analysis, TT genotype was related to DN susceptibility in Asians (TT vs Tt + tt: OR =2.21, 95% CI: 1.05–4.67, *p* = 0.04). The sensitivity analysis showed that the results in overall populations, Caucasians and Asians were dependable.

**Conclusions:**

No significant association was found among the ApaI, BsmI, FokI, TaqI polymorphisms and DN risk in overall populations, the TaqI variants might related to DN susceptibility in Asians. Further researches are required to testify our meta-analysis.

## Background

The vascular complications of diabetes mellitus (DM) can lead to high disability rates and mortality rates. Poorly controlled blood glucose leads to the occurrence and development of complications in patients with DM [1]. As one of the most serious diabetic microvascular diseases, diabetic nephropathy (DN) is the main reason of end-stage renal failure(ESRD) [2, 3]. Generally, DN is a multifactorial disease attribute to the interaction of environmental and genetic factors [4, 5]. Several factors always contributing to DN risk include abnormal renal hemodynamic responses, fatty acid metabolism caused by hyperglycemia, hypertension, and abnormal metabolism of vasoactive substances [6]. Recently, genetic predispositions have been found to play a key role in the development and progression of DN [7, 8]. Thus, searching for genetic markers for DN can identify patients who may benefit from prevention.

Previous studies showed that vitamin D endocrine system played an important role in the development of DM [9–11]. High levels of vitamin D can enhance pancreatic b-cell secretion functions and improve insulin resistance [12, 13]. There are accumulating evidences to suggest that vitamin D participated in a number of diseases such as DM and DN [14–16]. An increasing number of researches showed that there was a relationship between vitamin D receptor (VDR) gene polymorphisms and DN [17, 18]. Vitamin D exerts its functions by binding to the nuclear or cytosolic VDR, which serves as a transcription activator and is a member of the steroid / thyroid hormone receptor family [17]. At present, the most common studied single nucleotide polymorphisms (SNPs) of VDR are FokI (rs10735810), BsmI (rs1544410), ApaI (rs7975232) and TaqI (rs731236) [19, 20].

To provide an accurate estimation, we operated a systematic review to investigate the relation of DN and the four VDR gene polymorphisms(ApaI, BsmI, FokI and TaqI).

## Methods

### Search strategy

The related articles were searched from PubMed, Embase, Chinese National Knowledge Infrastructure and Chinese Wanfang databases. The The retrieval strategy were as follows: “VDR, vitamin D receptor, diabetic nephropathy (DN), ApaI, BsmI, FokI, TaqI, polymorphism, genotype”. In addition, we retrieved references of selected articles. Here are the inclusion criteria: 1) case-control study; 2)DN as ending; 3) carried out in human population; 4) Clearly define genetic variants of case and control can offer sufficient calculational data. Here are the exclusion criteria: (1) case reports or review articles; (2) articles that did not offer the detailed genotype information; (3) studies on the correlation between DN and other genes; (4) studies investigate the role of VDR genotype in other diseases. The largest or most recent study was selected when continuous or multiple publications that occur under the same search condition [21].

### Data extraction

For each study the following information was evaluated independently by two investigators according to the standard protocol:(1) name of first author; (2) year of publication; (3) original country; (4) ethnicity of the subjects; (5) genotype distribution or allele frequencies; and (6) number of cases and controls (Table 1). Genotypes for the VDR gene polymorphisms(ApaI, BsmI, FokI or TaqI) were designated with A, B, F and T for the absence of restriction sites and with a, b, f and t for their presence. In most studies, the ApaI, BsmI, FokI or TaqI genotype and allele frequency of the VDR gene were measured by PCR-restriction fragment length polymorphism (PCR-RFLP). In one study [22], pyrosequencing reactions were performed to determine the BsmI, FokI or TaqI genotype, Taqman 5′-nuclease assays were performed used to determine the ApaI genotype, the labels of FokI (C/T) are correspond to FokI (F/f),BsmI (A/G) are correspond to BsmI (B/b),ApaI (T/G) are correspond to ApaI (A/a),TaqI(T/C) are correspond to TaqI(T/t).

**Table 1:** Characteristics of the studies evaluating the effects of VDR BsmI,FokI, TaqI and ApaI gene polymorphisms on DN risk

Gene	Author	Ethnicity	Country	Type of DM	Sex	Case				Control				HWE(p)
sites	year				male/female									
BsmI						BB	Bb	bb	Total	BB	Bb	bb	Total	
	Bućan 2009	Caucasian	Croatia	DM1	—/—	1	8	5	14	6	18	9	33	0.566
	Martin 2009	Caucasian	Ireland	DM1	Case 359/296	106	321	228	655	111	325	238	674	0.998
					Control 286/388									
	Zhang 2012	Asian	China	DM2	Case 99/83 Control 68/54	3	57	122	182	0	26	96	122	0.188
FokI						FF	Ff	ff	Total	FF	Ff	ff	Total	
	Li 2005	Asian	China	DM2	30/64	9	17	13	39	28	22	5	55	0.821
	Bućan 2009	Caucasian	Croatia	DM1	—/—	4	6	4	14	9	18	6	33	0.566
	Martin 2009	Caucasian	Ireland	DM1	Case 359/296 Control 286/388	248	323	84	655	262	311	101	674	0.580
	Vedralová 2012	Caucasian	Czech	DM1/2	Case 75/57 Control 94/76	63	58	11	132	57	85	28	170	0.696
TaqI						TT	Tt	tt	Total	TT	Tt	tt	Total	
	Bućan 2009	Caucasian	Croatia	DM1	—/—	5	6	3	14	13	14	6	33	0.522
	Martin 2009	Caucasian	Ireland	DM1	Case 359/296 Control 286/388	103	327	225	655	98	327	249	674	0.575
	Nosratabadi 2010	Asian	Iran	DM2	Case 38/62 Control 41/59	9	55	36	100	4	63	33	100	0
	Han 2015	Asian	China	DM2	150/138	102	6	0	108	160	16	4	180	0
ApaI						AA	Aa	aa	Total	AA	Aa	aa	Total	
	Martin 2009	Caucasian	Ireland	DM1	Case 359/296 Control 286/388	185	323	147	655	200	322	152	674	0.303
	Nosratabadi 2010	Asian	Iran	DM2	Case 38/62 Control 41/59	9	64	27	100	9	63	28	100	0.002
	Zhang 2012	Asian	China	DM2	Case 99/83 Control 68/54	19	89	74	182	11	65	46	122	0.075
	Han 2015	Asian	China	DM2	150/138	2	50	56	108	18	80	82	180	0.814

### Statistical analysis

The odds ratio (OR) with 95% confidence interval (CI) were used to evaluate the association among the four VDR gene polymorphisms and DN risk. Statistical analysis were divided into Caucasian and Asian populations in subgroup analysis. The Chi-square based Q-statistic test was used to assess the heterogeneity between the studies. *P* < 0.10 indicated there is a significant heterogeneity among the studies. If the *P* value was no more than 0.1, pooled OR was estimated using a random-effect model using the DerSimonian and Laird method (D + L), otherwise a fixed-effect model using the Mantel–Haenszel (M–H) method was carried out. The asymmetry funnel plots were used to estimate the publication bias. The exact test was carried out to assess whether the genotype distribution in control population were accord with Hardy-Weinberg equilibrium (HWE) expectations. A sensitivity analysis of the overall population was used by omitting one study in each turn. Cochrane Review Manager Version 5.1 (Cochrane Library, Oxford, UK) was used to performed the statistical analyses. All the *P* values were double tailed test and the significance was set at *P* < 0.05.

## Results

### Characteristics of the studies

**Fig. 1:**
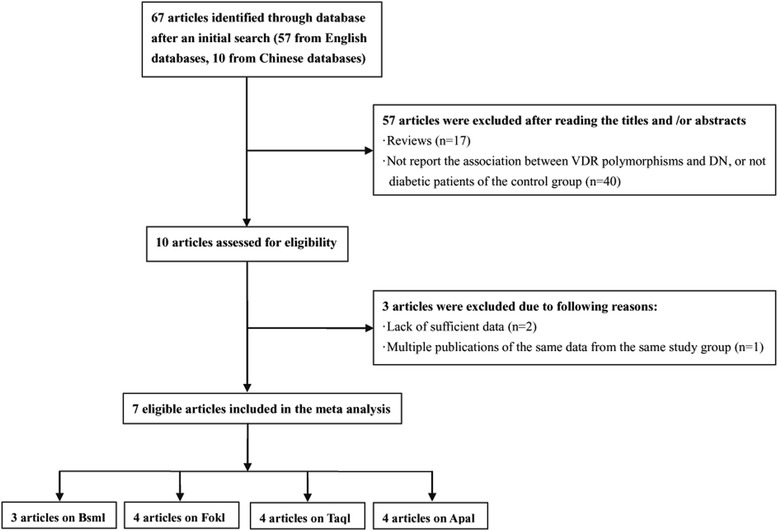
Flow diagram of included and excluded studies

The search yielded 67 articles, 7 studies [18, 22–27] contain 1230 DN patients and 1334 diabetic controls were finally recruited into our meta-analysis according to the inclusion and exclusion criteria, all studies reporting the association among ApaI, BsmI, FokI, TaqI of VDR gene polymorphisms and DN susceptibility (Fig. 1). The main characteristics of these selected studies were summarized in Table 1, including the first author’s name, year of publication, original country and genotype distribution.

### Quantitative data synthesis

**Fig. 2:**
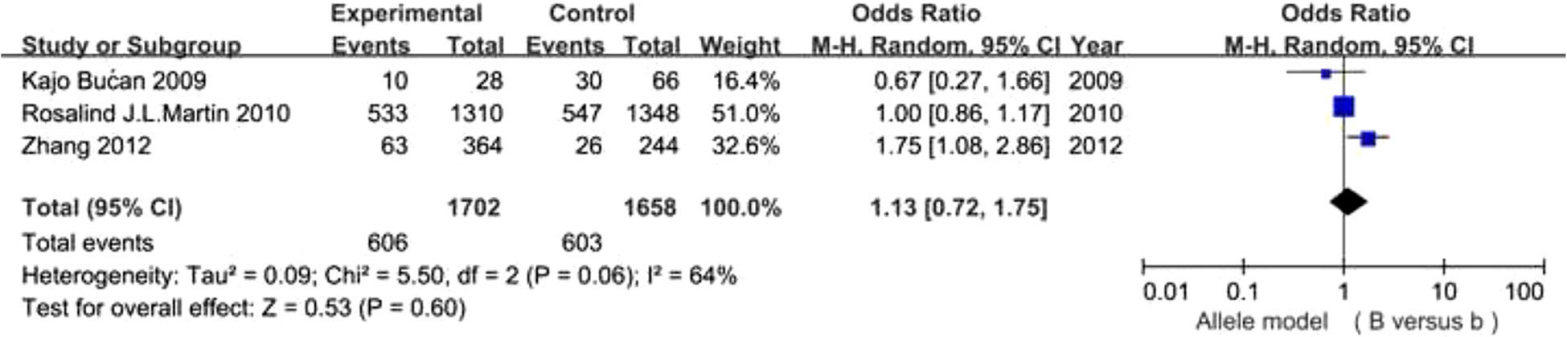
Association of VDR BsmI gene polymorphism(B vs b)with DN susceptibility in overall populations

**Fig. 3:**
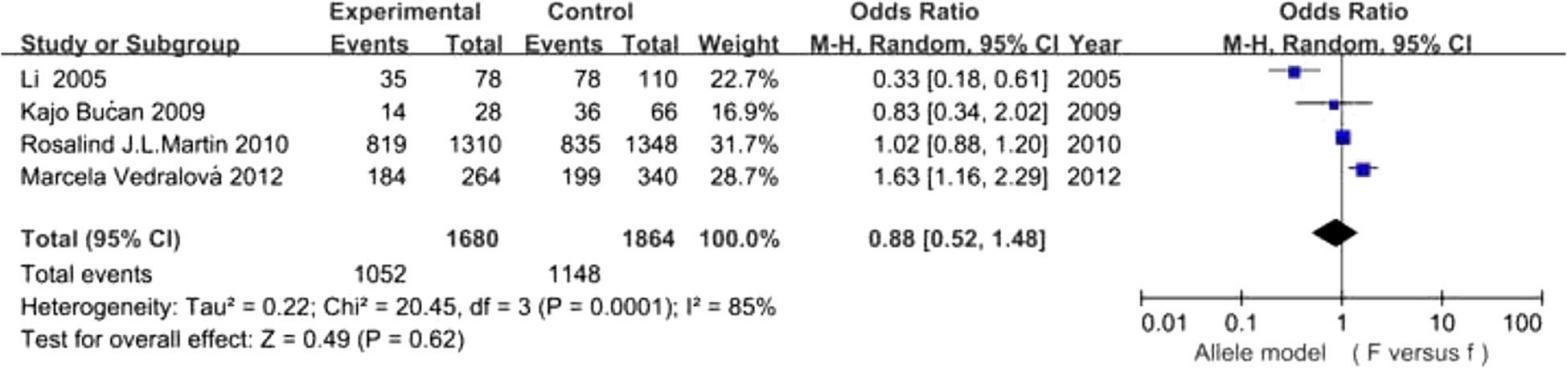
Association of VDR FokI gene polymorphism(F vs f) with DN susceptibility in overall populations

**Fig. 4:**
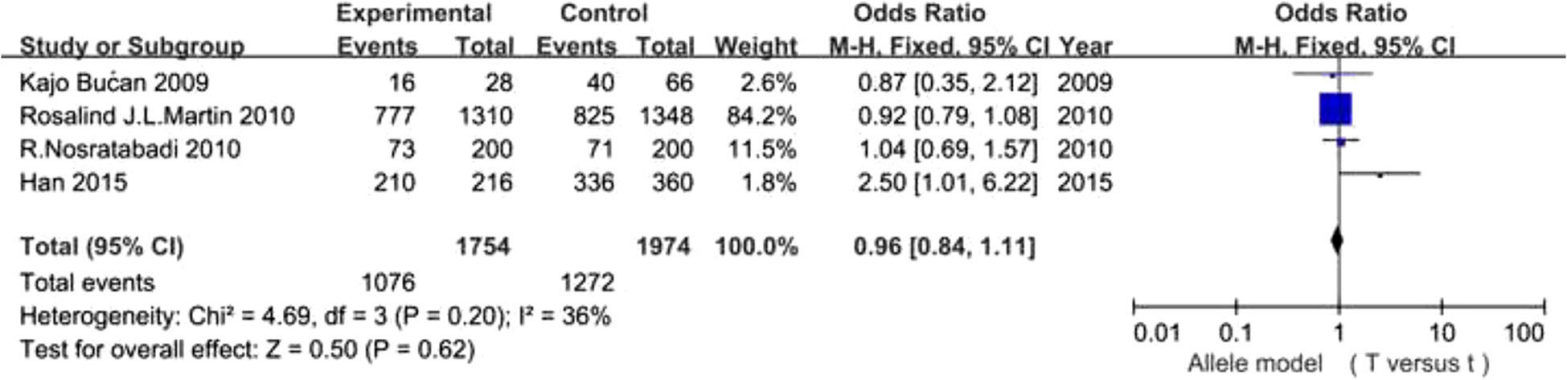
Association of VDR TaqI gene polymorphism(T vs t) with DN susceptibility in overall populations

**Fig. 5:**
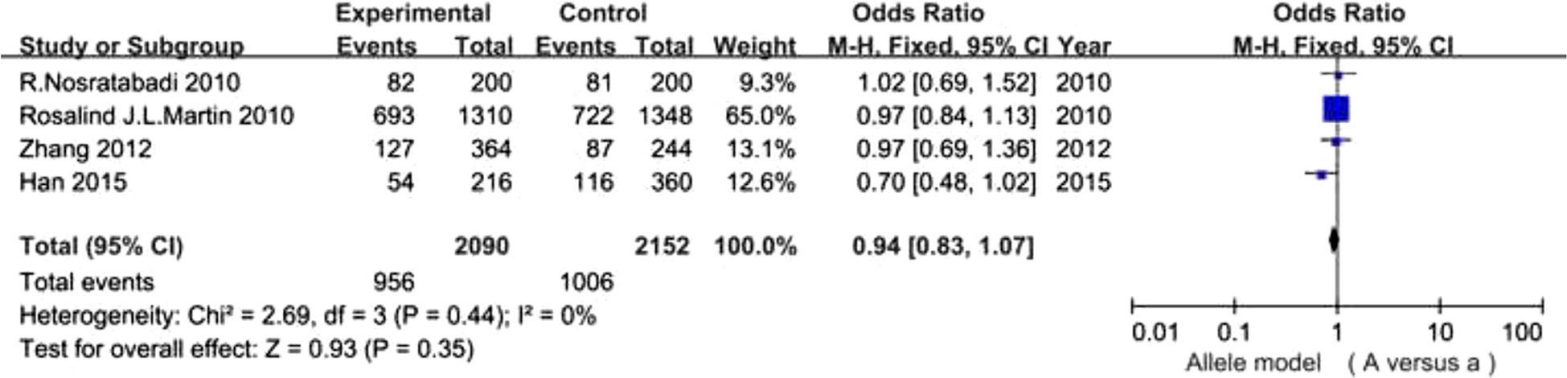
Association of VDR ApaI gene polymorphism(A vs a) with DN susceptibility in overall populations

It has been shown in Table 2 that the risk for DN conferred by VDR gene polymorphisms did not show significant difference in the overall 7 studies (all *P* values >0.05) (Figs. 2, 3, 4 and 5).In the stratified analysis, there was a relationship between TT genotype and DN risk in Asians in only two studies of 488 patients(TT vs Tt + tt: OR =2.21, 95% CI: 1.05–4.67, *p* = 0.04), however, *p* value equals to 0.04 seems not enough to support relationship between genetic variant and diabetic nephropathy. Besides, there was no significant correlation between other three polymorphisms and DN risk in Asians and Caucasians(Table 2). Altogether, the results of the meta-analysis can be described as negative. Particularly worth mentioning is that only one published study in Asian populations was included in the BsmI and FokI groups, the subgroup for Asians might not be reliable in these two groups. Two studies [24, 27] were excluded because the genotype distributions in controls were obviously deviated from the HWE. Sensitivity analysis showed that the results were dependable for overall populations, Caucasians and Asians.

**Table 2:** Meta analysis of the association of VDR BsmI,FokI, TaqI and ApaI gene polymorphisms with DN risk

Genetic	Group and	Studies	Q test p	Model	OR(95%CI)	p
contrasts	subgroups	number	value	selected		
BsmI						
B vs b	Overall	3	0.06	Random	1.13 [0.72, 1.75]	0.60
	Caucasian	2	0.39	Fixed	0.99 [0.85, 1.16]	0.93
	Asian	1	-	Fixed	1.75 [1.08, 2.86]	0.02
BB vs Bb + bb	Overall	3	0.38	Fixed	0.98 [0.74, 1.30]	0.89
	Caucasian	2	0.36	Fixed	0.96 [0.72, 1.28]	0.77
	Asian	1	-	Fixed	4.78 [0.24, 93.31]	0.30
bb vs BB + Bb	Overall	3	0.11	Random	0.84 [0.53, 1.33]	0.45
	Caucasian	2	0.55	Fixed	0.99 [0.79, 1.24]	0.93
	Asian	1	-	Fixed	0.55 [0.32, 0.94]	0.03
FokI						
F vs f	Overall	4	0.0001	Random	0.88 [0.52, 1.48]	0.62
	Caucasian	3	0.04	Random	1.19 [0.81, 1.73]	0.38
	Asian	1	-	Fixed	0.33 [0.18, 0.61]	0.0004
FF vs Ff + ff	Overall	4	0.004	Random	0.92 [0.50, 1.71]	0.80
	Caucasian	3	0.05	Random	1.24 [0.74, 2.05]	0.41
	Asian	1	-	Fixed	0.29 [0.12, 0.72]	0.008
ff vs FF + Ff	Overall	4	0.005	Random	1.18 [0.52, 2.72]	0.69
	Caucasian	3	0.18	Fixed	0.78 [0.59, 1.03]	0.08
	Asian	1	-	Fixed	5.00 [1.61, 15.56]	0.005
TaqI						
T vs t	Overall	4	0.20	Fixed	0.96 [0.84, 1.11]	0.62
	Caucasian	2	0.89	Fixed	0.92 [0.79, 1.08]	0.30
	Asian	2	0.09	Random	1.46 [0.63, 3.38]	0.37
TT vs Tt + tt	Overall	4	0.15	Fixed	0.97 [0.79, 1.20]	0.77
	Caucasian	2	0.95	Fixed	0.89 [0.71, 1.11]	0.31
	Asian	2	0.89	Fixed	2.21 [1.05, 4.67]	0.04
tt vs TT + Tt	Overall	4	0.68	Fixed	1.08 [0.83, 1.40]	0.56
	Caucasian	2	0.89	Fixed	1.10 [0.82, 1.48]	0.52
	Asian	2	0.22	Fixed	1.01 [0.58, 1.77]	0.97
ApaI						
A vs a	Overall	4	0.44	Fixed	0.94 [0.83, 1.07]	0.35
	Caucasian	1	-	Fixed	0.97 [0.84, 1.13]	0.73
	Asian	3	0.33	Fixed	0.89 [0.72, 1.10]	0.26
AA vs Aa + aa	Overall	4	0.14	Fixed	0.89 [0.72, 1.11]	0.31
	Caucasian	1	-	Fixed	0.93 [0.74, 1.18]	0.57
	Asian	3	0.06	Fixed	0.73 [0.43, 1.23]	0.24
aa vs AA + Aa	Overall	4	0.79	Fixed	1.06 [0.87, 1.28]	0.58
	Caucasian	1	-	Fixed	0.99 [0.77, 1.29]	0.96
	Asian	3	0.75	Fixed	1.14 [0.85, 1.53]	0.37

### Publication bias

Begg’s funnel plots and Egger’s test were carried out to analyse the publication bias. Results showed that there was no evidence of publication bias in the 7 studies (data not shown). However, due to the small number of studies, publication bias cannot be ruled-out in this paper.

## Discussion

DN is the most common microvascular complication in diabetes patients, and the most common reason of ESRD in the United States [28]. 40% DM patients suffer from DN, and the occurrence have no association with hyperglycemia, suggesting genetic factors may be involved in the development of DN [29, 30].Currently, VDR has also been illustrated to be associated with the occurrence and development of DN [31].

The vitamin D has important biological functions, such as modulating immunity system, influencing insulin secretion and improving insulin resistance [32, 33], which are involved in the etiology of DN and more likely to be influenced by VDR gene polymorphisms. Given the controversial results from published individual studies with small sample sizes, we carried out a meta-analysis to clarify the association between the VDR gene polymorphisms and susceptibility to DN.

The VDR gene is located on chromosome 12 at q12-q14, it has14eons, 8 protein-coding exons and 6 untranslated region. ApaI and BsmI VDR gene variants locate in intron 8 and TaqI variants locate in exon 9. The FokI variants locate in the codon initiating translation, which has been reported to cause severe protein dificiency [25]. A large amount of common chronic disorder diseases, such as type 1 and type 2 DM, have been found to be relevant to specified VDR gene polymorphisms [34]. The effects of VDR gene polymorphisms on gene expression are still not clearly understood. VDR gene variants may have effect on RNA translational efficiency [35]. Distinctions of VDR mRNA express level and stability can affect the activity of renin-angiotensin system and participate in the progress of DN [26, 27].

In this study, we analyzed the VDR gene functional polymorphisms (ApaI, BsmI, FokI and TaqI) as potential genetic markers for DN. Nevertheless, the results of our research showed that none of these polymorphisms was significantly associated with DN risk in diabetic patients in overall population(all *P* values >0.05). Since genetic factors can affect the results of meta analysis, subgroup analysis were also preformed by race for the four polymorphisms. Our results indicate that there was a relationship between TT genotype and DN risk in Asians (TT vs Tt + tt: OR =2.21, 95% CI: 1.05–4.67, *p* = 0.04), however, *p* value equals to 0.04 seems not enough to support relationship between genetic variants and DN. Since only one published study in Asian populations was included in the BsmI and FokI groups, the stratified analysis for Asians might not be reliable in these two groups. Besides, there was no significant associations among the other three gene variants and DN in Caucasians and Asians(Table 2). Altogether, the results of the meta-analysis can be described as negative. Liu [34] reported that the FokI gene variants was related to DN susceptibility in Caucasians. We have a different result, and we performed a more comprehensive research on the association among four gene polymorphisms (ApaI, BsmI, FokI and TaqI) of VDR and DN risk. However, further large-scale studies should be carried out to testify our research.

For better understanding of the results, several limitations in our study should be mentioned. First, the number of subjects were relatively small, so we lacked sufficient statistical power to estimate the associations between VDR gene variants and susceptibility to DN. Second, meta-analysis is a retrospective study that may cause selection bias, which may have an impact on the reliability of results. Third, our study failed to acquire raw data from the selected articles, which may lead to further limitation in the assessment of potential effects of VDR gene polymorphisms in the occurrence and progression of DN.

Our research provide evidence that VDR TaqI polymorphism may be relevant to the risk of DN in DM patients among Asians. However, the number of articles included is really small, which is an important limit, and with such a small number of studies, subgroup (Asian/Caucasian) results we reported have to be considered as exploratory. Martin et al.’s [22] is by far the largest sample size study in our research. Consequently, the overall meta-analysis report results very close to those of Martin et al. Therefore, large-scale studies are still needed to provide a more representative meta-analysis.

## Conclusions

In conclusion, our meta-analysis showed that no significant association was observed among the ApaI, BsmI, FokI and TaqI variants and DN susceptibility in overall populations, the TaqI variants may be associated with DN risk in Asian populations. Future larger scale epidemiological investigation of this topic should be conducted to confirm or refute our findings.

## Abbreviations

CI: Confidence interval
D + L: DerSimonian and Laird method
DM: Diabetes mellitus
DN: Diabetic nephropathy
ESRD: End-stage renal failure
HWE: Hardy-Weinberg equilibrium
M–H: Mantel–Haenszel.
OR: Odds ratio
SNPs: Single nucleotide polymorphisms
VDR: Vitamin D receptor
